# Supplementary material for: Large-scale gene losses underlie the genome evolution of parasitic plant Cuscuta australis
Source: Nat Commun. 2018 Jul 11;9:2683. doi: 10.1038/s41467-018-04721-8 (PMC6041341; doi:10.1038/s41467-018-04721-8)
Supplement: Supplementary file 1 — Supplementary Information [file 41467_2018_4721_MOESM1_ESM.pdf]

Title: Large-scale gene losses underlie the genome evolution of parasitic plant *Cuscuta australis*

Sun, et al.

Description of Supplementary Files

File Name: Supplementary Information

Description: Supplementary Figures, Supplementary Tables, Supplementary Notes and Supplementary References

File Name: Supplementary Data 1

Description: Supplementary Data 1a. Gene family expansion and contraction;

Supplementary Data 1b. Number of orthogroup genes in each species;

Supplementary Data 1c. Gene loss identified using Arabidopsis genes as reference;

Supplementary Data 1d. Tissue-specific expression levels of the orthogroup genes in *Solanum lycopersicum*, *Ipomoea nil*, *and Cuscuta pentagona*

File Name: Supplementary Data 2

Description: Supplementary Data 2a. GO enrichment of the orthogroups whose orthologous members are conserved in 7Ref-Species but lost in both *Cuscuta australis* and *Utricularia gibba*. Supplementary Data 2b. GO enrichment of the orthogroups whose orthologous members are conserved in 7Ref-Species and *Utricularia gibba*, but lost in *Cuscuta australis*. Supplementary Data 2c. GO enrichment of the orthogroups whose orthologous members are conserved in 7Ref-Species and in *Cuscuta australis*, but lost in *Utricularia gibba*. Supplementary Data 2d. GO enrichment of the principally expressed genes in *Cuscuta australis* haustoria. Supplementary Data 2e. GO enrichment of positively selected genes in *Cuscuta australis*. Supplementary Data 2f. GO enrichment of relaxed purifying selection genes in *Cuscuta australis*. Supplementary Data 2g. GO enrichment of genes in expanded gene families in *Cuscuta australis*.

File Name: Supplementary Data 3

Description: Supplementary Data 3a. Functional annotations of pseudogenes.

Supplementary Data 3b. R genes in *Cuscuta australis* and 7Ref-Species.

Supplementary Data 3c. TPS genes in *Cuscuta australis* and 7Ref-Species.

Supplementary Data 3d. P450 genes in *Cuscuta australis* and 7Ref-Species.

Supplementary Data 3e. RLK genes in *Cuscuta australis* and 7Ref-Species.

Supplementary Data 3f. Functional annotations of principally expressed genes in *Cuscuta australis* haustoria.

File Name: Supplementary Data 4

Description: Presence/absence of plastid tRNA, rRNA, and protein-coding genes in *Nicotiana tabacum*, *Ipomea nil*, four *Cuscuta spp*., and two root parasitic plants *Striga hermonthica* and *Orobanche cumana*.

File Name: Supplementary Data 5

Description: Copy numbers of Copia and Gypsy families in *Cuscuta australis* and *Ipomoea nil*.

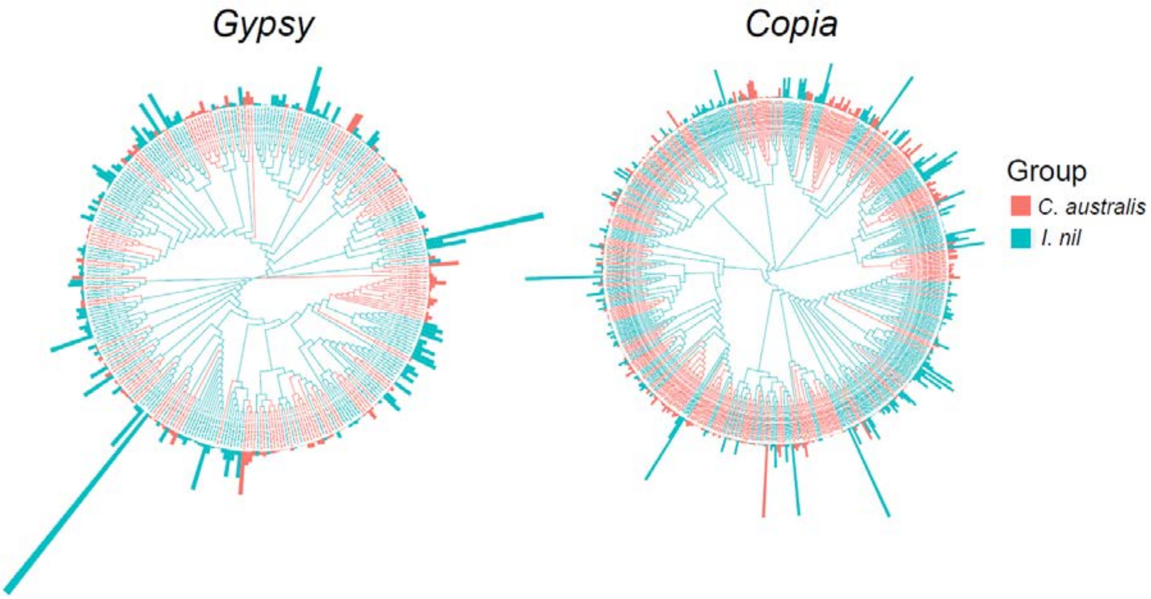

Supplementary Figure 1. Phylogeny of *LTR/Gypsy* and *LTR/Copia* in *Cuscuta australis* and *Ipomoea nil*. Branches of *Cuscuta australis* and *Ipomoea nil* were denoted as red and blue respectively. Bars outside dendrogram denote copy numbers of the repetitive family (detailed copy numbers can be found in Supplementary Data 5).

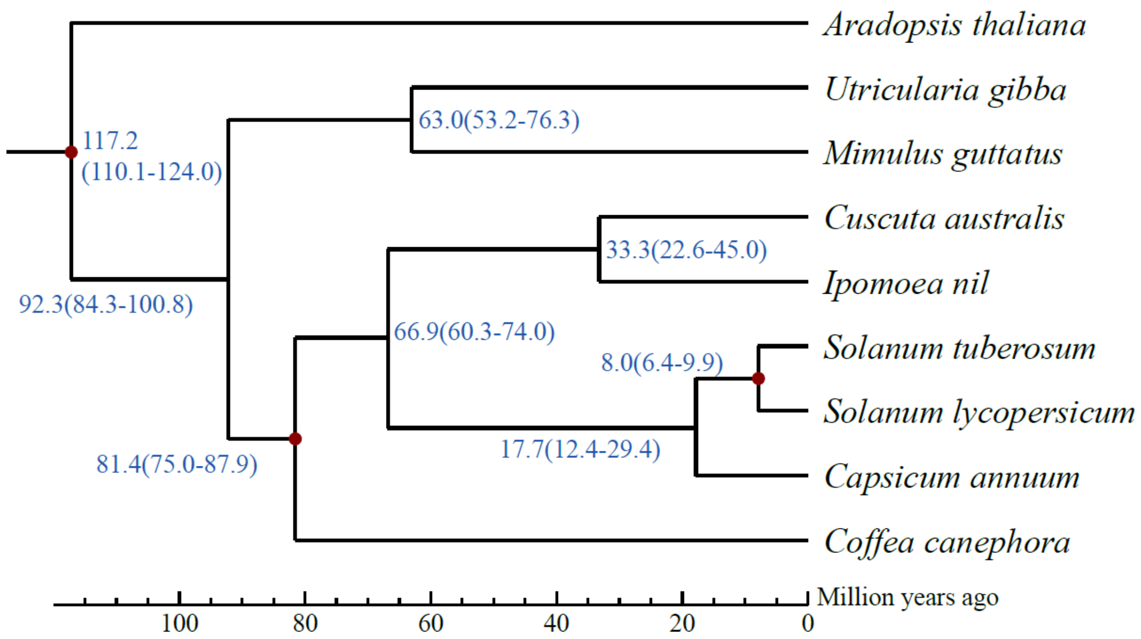

Supplementary Figure 2. Divergence time estimation. Divergence time was estimated using the mcmctree embedded in the PAML package. The most likely divergence times from the most recent comment ancestor are given along each node, and the estimated ranges of divergence times are shown in the parentheses. Numbers in the parentheses are the predicted divergence times (95% confident intervals). The node dots indicate the time of speciation retrieved from the Timetree database (http://timetree.org), which were used for calibrating the divergence estimation.

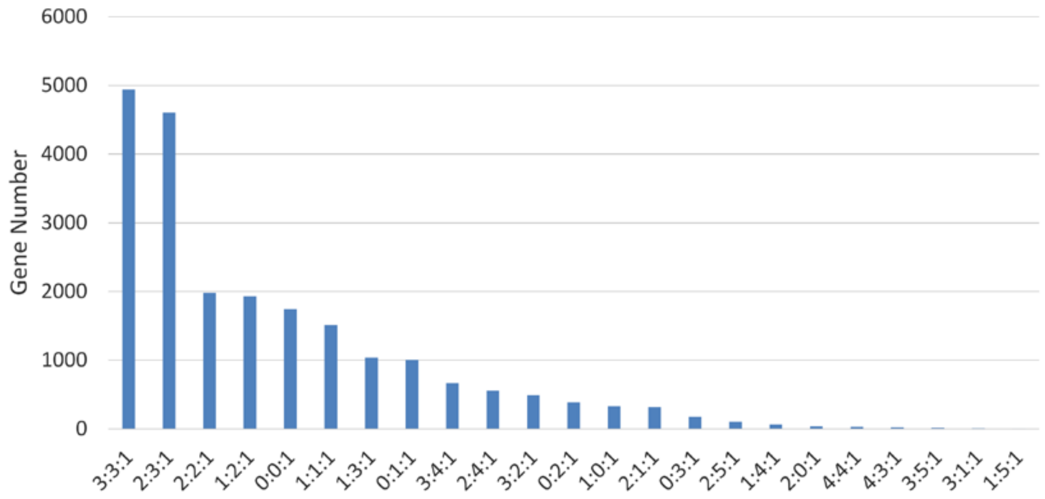

Supplementary Figure 3. Statistics of different patterns of depths of *Cuscuta australis* and *Ipomoea nil* syntenic blocks covering all *Coffea canephora* syntenic orthologs. Every syntenic block was anchored to the chromosomes of *Coffea canephora*, and the numbers of the syntenic blocks from *Cuscuta australis* and *Ipomoea nil* covering each individual *Coffea canephora* syntenic gene were determined to obtain the syntenic coverage information, which is described by “number of syntenic block of *Cuscuta australis*:number of syntenic block of *Ipomoea nil*:1” for each *Coffea canephora* gene locus. The syntenic *Coffea canephora* genes with a specific coverage pattern were counted and plotted.

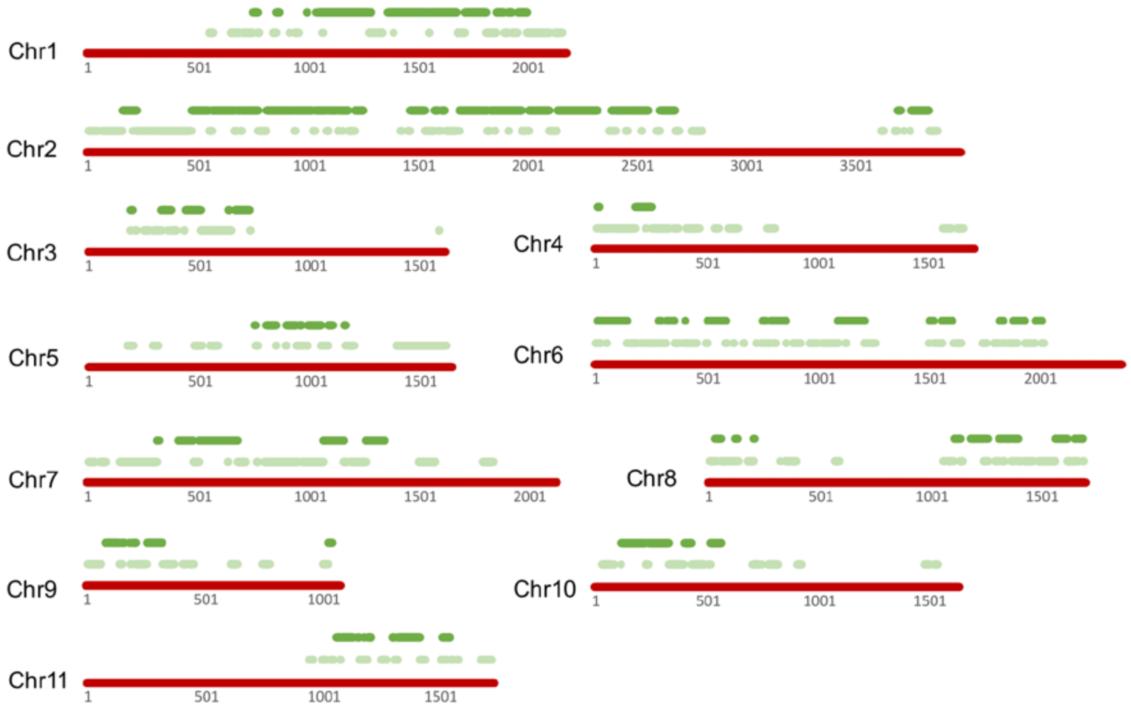

Supplementary Figure 4. Distribution of *Coffea canephora* genes having syntenic coverages of 3:3:1 or 2:3:1 from the *Cuscuta australis* and *Ipomoea nil* syntenic blocks over the *Coffea canephora* chromosomes. *Coffea canephora* genes having syntenic coverages 3:3:1 from *Cuscuta australis* and *Ipomoea nil* syntenic blocks are plotted as green dots, and those having 2:3:1 coverages are plotted as light green dots.

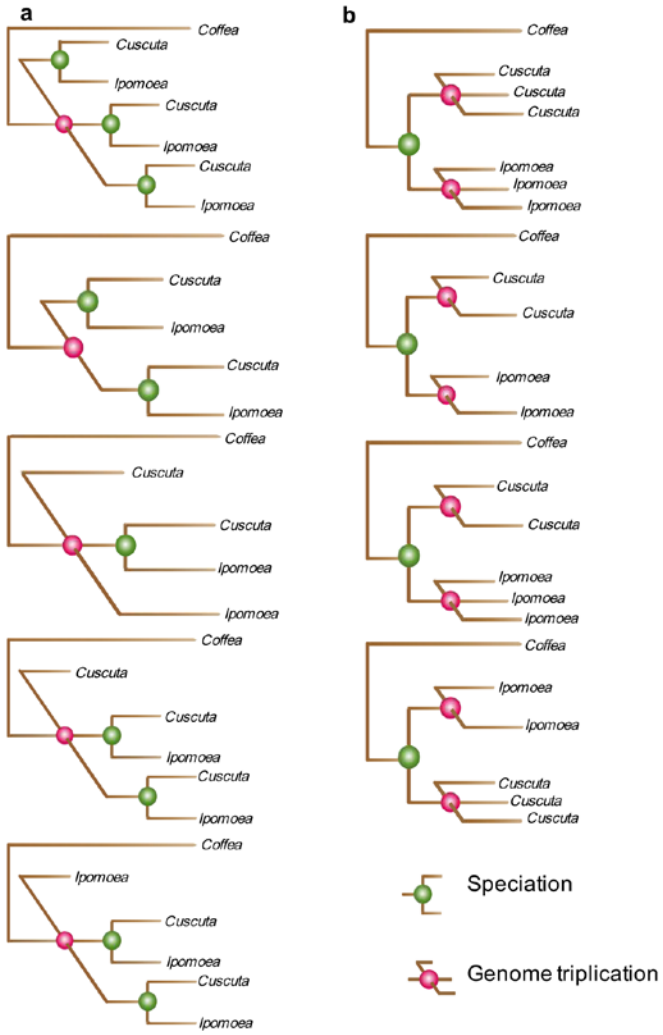

Supplementary Figure 5. Topologies of gene trees depicting the possible scenarios of speciation among *Cuscuta australis*, *Ipomoea nil*, and *Coffea canephora*. Orthogroups that contain one *Coffea canephora* gene, at least two *Cuscuta australis* orthologs, and at least two *Ipomoea nil* orthologs were extracted. All possible topologies were summarized after reconstructing the phylogenetic gene trees of the sequences in an orthogroup. **a.** One possible speciation scenario, in which WGD happened before the speciation between *Ipomoea* and *Cuscuta*. **b.** The alternative speciation hypothesis in which WGD happened independently after the speciation between *Ipomoea* and *Cuscuta*.

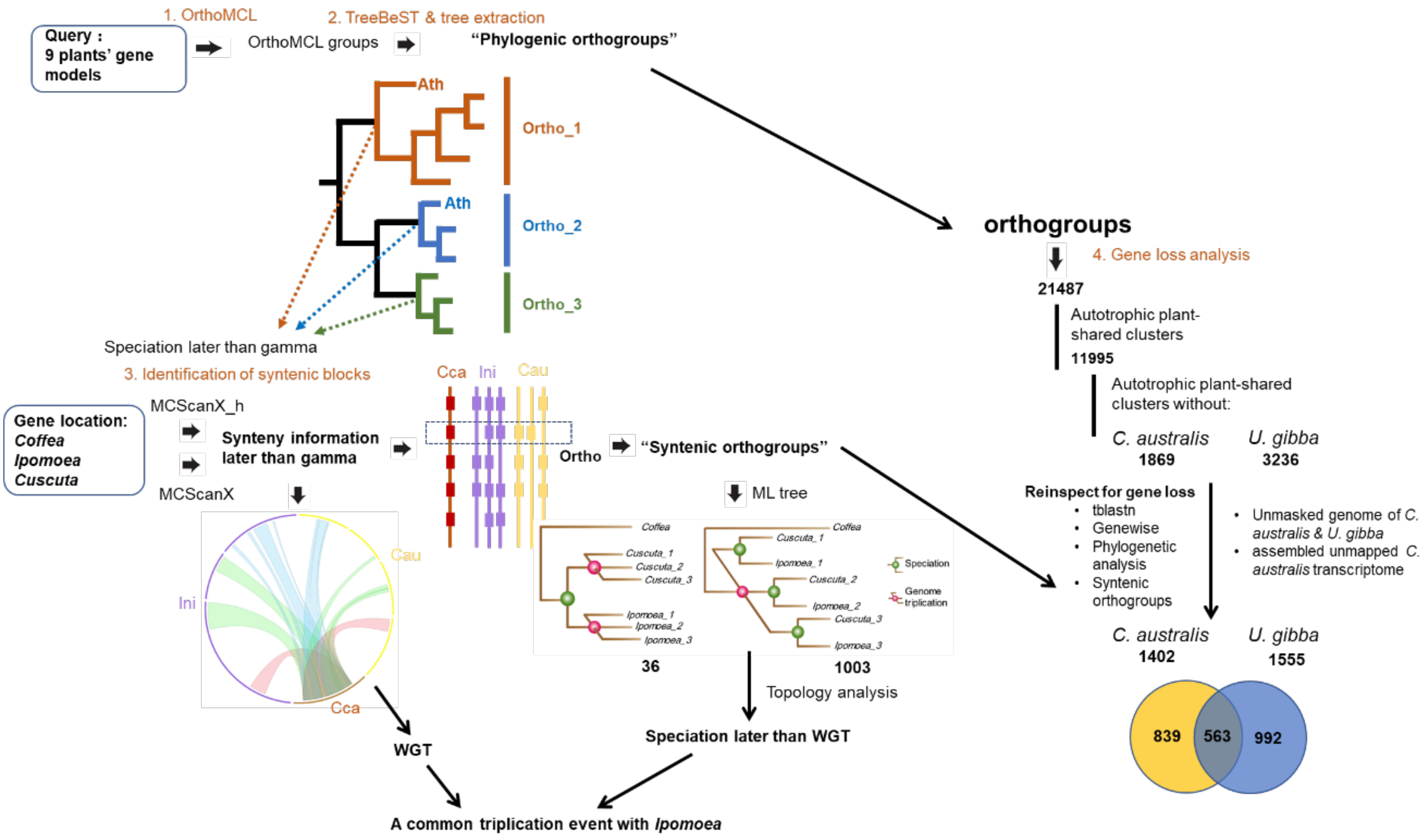

1. OrthoMCL
2. TreeBeST & tree extraction
Query : 9 plants' gene models
OrthoMCL groups
"Phylogenic orthogroups"
Ath
Ortho_1
Ath
Ortho_2
Ortho_3
Speciation later than gamma
3. Identification of syntenic blocks
Cca
Ini
Cau
Gene location: Coffea Ipomoea Cuscuta
MCScanX_h
Synteny information later than gamma
Ortho
"Syntenic orthogroups"
MCScanX
ML tree
Cau
Ini
Cca
Coffea
Cuscuta_1
Cuscuta_2
Cuscuta_3
Ipomoea_1
Ipomoea_2
Ipomoea_3
Speciation
Genome triplication
36
1003
Topology analysis
WGT
Speciation later than WGT
A common triplication event with Ipomoea
orthogroups
4. Gene loss analysis
21487
Autotrophic plant-shared clusters
11995
Autotrophic plant-shared clusters without:
C. australis
1869
U. gibba
3236
Reinspect for gene loss
tblastn
Genewise
Phylogenetic analysis
Syntenic orthogroups
Unmasked genome of C. australis & U. gibba
assembled unmapped C. australis transcriptome
C. australis
1402
U. gibba
1555
839
563
992

Supplementary Figure 6. Pipeline of orthogroup identification and syntenic analysis. Analysis pipeline indicating the steps of identification of orthogroups and syntenic blocks. The gene models of *Cuscuta australis* and 7Ref-Species were clustered using OrthoMCL (Step 1), and resulted groups were fed to TreeBeST[1] (v1.9.2) for tree extraction (Step 2), and the "phylogenetic orthogroups" were obtained. MCScanX_h and MCScanX were employed to generate the information of the syntenic blocks (derived from speciation and gene duplication after the gamma event) in *Cuscuta australis* and the reference species (Step 3), and the information of syntenic blocks were used to obtain the "syntenic orthogroups". The "phylogenetic orthogroups" were used to analyze gene loss and "syntenic orthogroups" were used to reinspect gene loss (Step 4). The information of syntenic blocks was used for analyzing the recent WGD in *Cuscuta*, *Ipomoea*, and *Coffea*, to confirm whether whole-genome triplication (WGT) happened during the evolution of *Cuscuta* and *Ipomoea*. The phylogenetic relationship of the orthologs among *Cuscuta australis*, *Ipomoea nil*, and *Coffea canephora* were examined to conclude the order of WGT and speciation between *Cuscuta* and *Ipomoea*. had a common whole-genome triplication was concluded. Based on these lines of evidence, that *Cuscuta* and *Ipomoea* had a common WGT event before the split from their common ancestor was concluded.

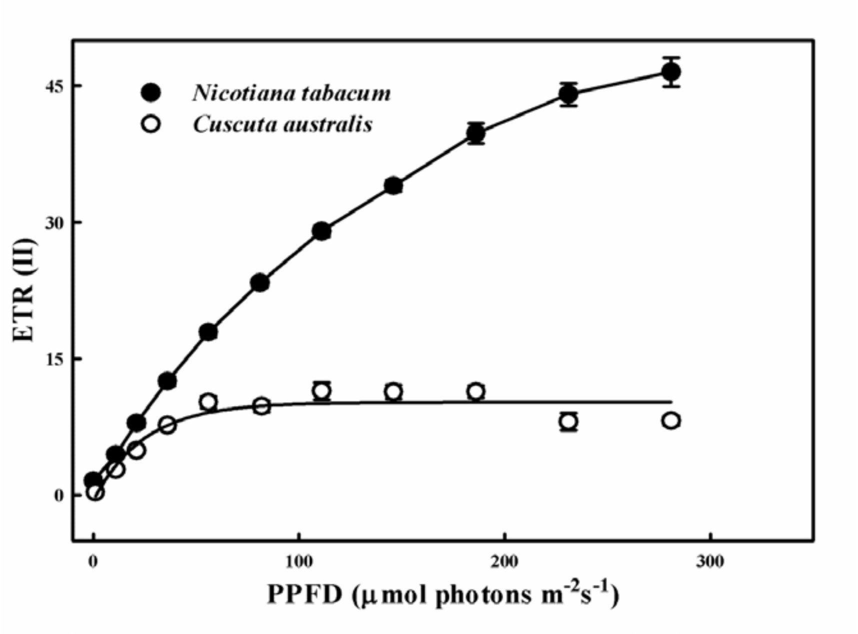

Supplementary Figure 7. Light-response changes in photosynthetic electron flow through PSII (ETR(II)) in *Cuscuta australis* stems and leaves of *Nicotiana tabacum*.

Data are means ± SE (n = 5).

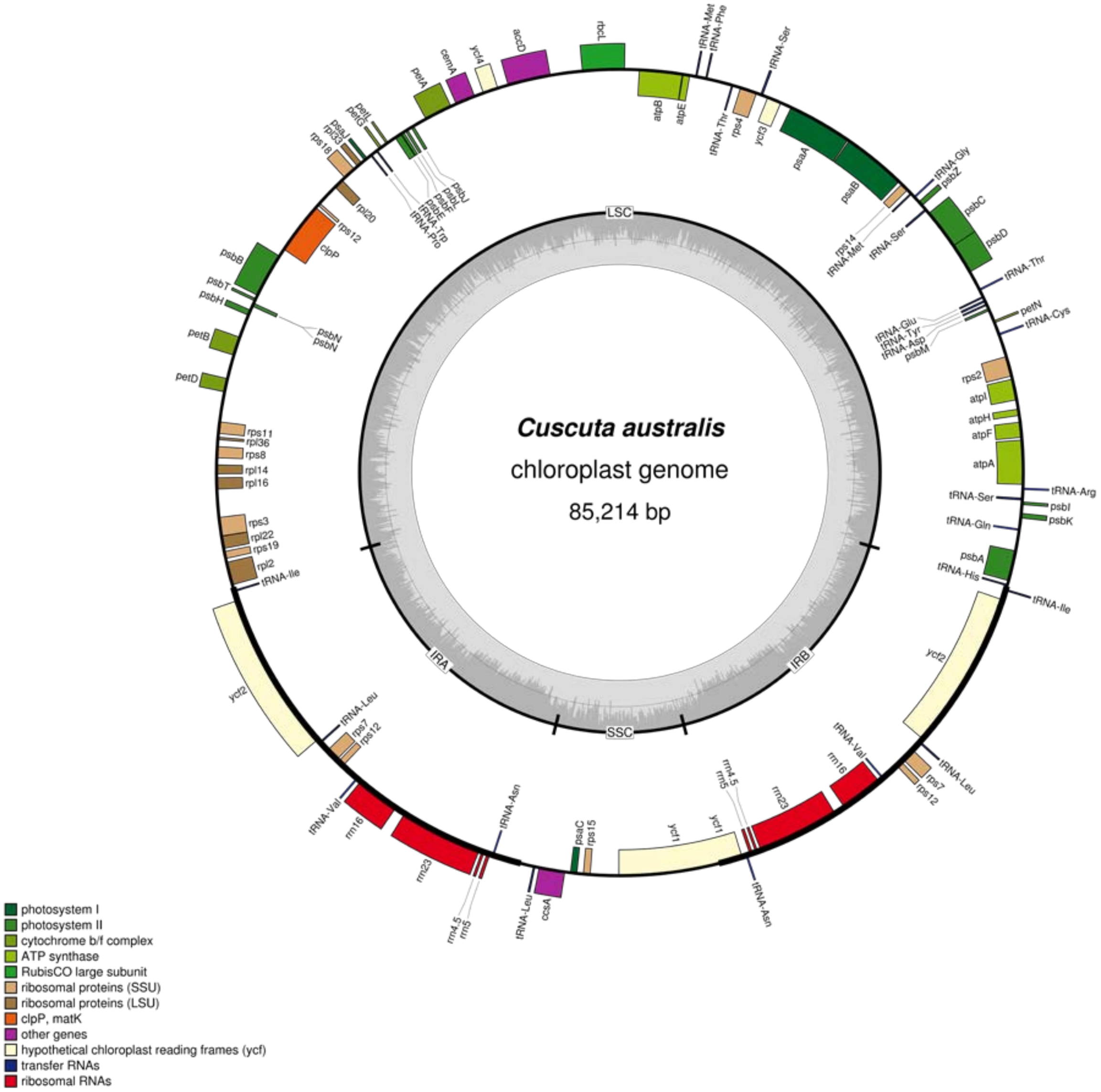

Supplementary Figure 8. Circular map of the complete plastid genome of *Cuscuta australis.*

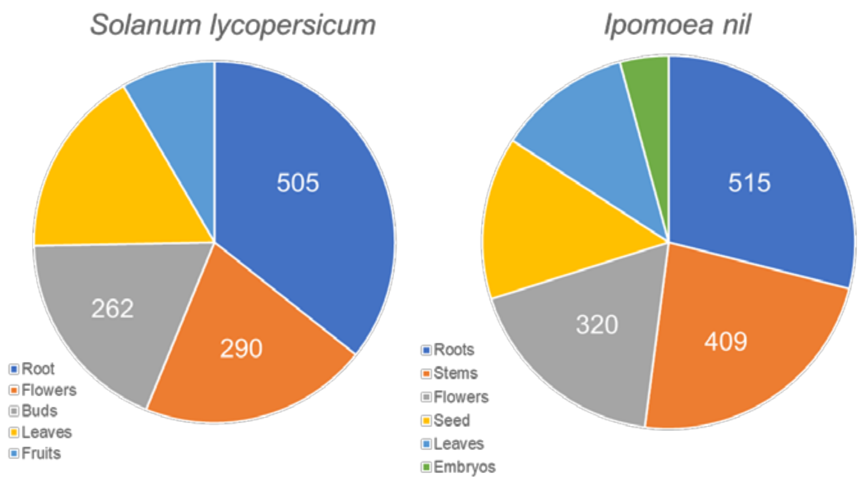

Supplementary Figure 9. The principally expressed tissues (PETs) of *Cuscuta. australis* haustorium principally expressed genes' orthologues in *Solanum lycopersicum* and *Ipomoea. nil*. The respective PETs of the orthogroups, which have principally expressed genes in *C. australis* haustoria were identified in *Solanum lycopersicum* and *Ipomoea. nil*. The numbers of orthogroups, which have PETs in leaves, roots, flowers, and other tissues, are shown in the respective slices of the pie charts. Only the slices of the top three PETs are shown with numbers.

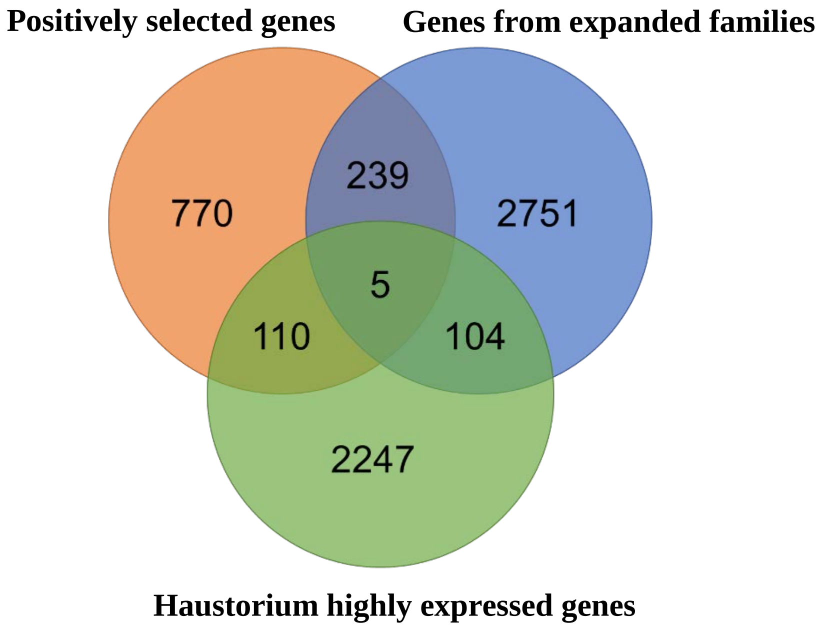

Supplementary Figure 10. Venn diagram of the positively selected genes, genes from the expanded gene families, and genes highly expressed in prehaustoria/haustoria.

Supplementary Table 1. Genome assembly statistics of *Cuscuta australis*

Type	Contigs		Scaffolds		Superscaffolds	
	Size	Number	Size	Number	Size	Number
N90	904385	87	1850081	46	1850081	46
N80	1418971	64	2904980	34	2904980	34
N70	2100488	48	3950442	27	3950442	27
N60	2542181	36	4610076	21	4610076	21
N50	3625894	27	5945234	16	5945234	16
N40	4249895	20	7261444	12	7261444	12
N30	4403227	14	7763232	8	7763232	8
N20	4855865	8	10192992	5	10192992	5
N10	6540893	4	10743034	3	10743034	3
Total Length	266740251	249	267213323	103	267213323	103
Maximum Length	10192992	-	13922953		13922953	
Minimum Length	5448	-	16649		16649	

Supplementary Table 2. Contigs containing organellar sequences

	Contig_ID	Length	Average depth	Note
Plastid	C141C	85214	2593.871433	complete plastome
	C131M	186399	1214.601551	
	C135M	146508	1685.295412	
	C136M	141688	1072.910925	
	C139M	134523	1862.792701	
	C140M	123029	1711.995977	
	C143M	97161	1609.256254	
	C146M	87099	1842.503	
	C149M	81803	1723.384704	
	C154M	77475	1336.538172	
	C162M	57724	1447.579904	
	C173M	50918	1344.030801	
	C175M	50434	778.2785238	
Mitochondria	C176M	50271	1563.827082	
	C185M	48206	1842.295907	
	C186M	45851	1500.950283	
	C189M	45272	928.8866392	
	C192M	43229	1163.849815	
	C193M	43010	1248.797399	
	C196M	41178	1140.851044	
	C202M	39887	1245.670002	
	C208M	36278	1340.751504	
	C220M	33415	1163.144485	
	C221M	33114	1031.367691	
	C222M	32823	962.1748523	
	C225M	29664	1132.485868	

C235M	22150	582.2652773
C238M	18912	1009.900755
C246M	14731	1096.597367
C253M	8634	832.8695944

Supplementary Table 3. Accuracy and heterozygosity of *Cuscuta australis* genome assembly

**Errors in Pacbio assembly before Pilon correction**	
Numbers of homozygous SNPs	24636
Numbers of homozygous INDELs	115496
Estimated accuracy	0.9995
**Errors in Pacbio assembly after Pilon correction**	
Numbers of homozygous SNPs	23656
Numbers of homozygous INDELs	13671
Estimated accuracy	0.9999
**Within genome heterozygosity**	
Within genome heterozygous SNPs	29070
Numbers of heterozygous INDELs	4733
Estimated within genome heterozygosity	0.013%

Supplementary Table 4. Repetitive elements in *Cuscuta australis* genome

	Ipomoea nil			*Cuscuta australis*		
**Classes**	**Copies**	**Total size**	**Percentage in genome**	**Copies**	**Total size**	**Percentage in genome**
DNA Transposon	237,062	79,245,270	10.8%	73,415	19,401,328	7.3%
LTR	191,314	172,365,468	23.5%	79,011	63,746,515	23.9%
LINE	28,561	19,757,447	2.7%	18,039	6,456,063	2.4%
SINE	25,188	6,566,780	0.9%	8,131	1,239,886	0.5%
Helitron	7,778	4,266,513	0.6%	7,138	2,852,074	1.1%
Simple Repeats	366,864	22,582,319	3.1%	85,340	6,672,582	2.5%
Unknown	623,410	181,060,611	24.6%	259,895	62,796,207	23.5%
Total	-	474,203,812	64.5%	-	155,040,159	58%

Supplementary Table 5. Two cluster analyses of *Cuscuta australis* and related species.

Outgroup	Ingroup A	Ingroup B	bA	bB	delta	s.e.	Z	CP	Faster
Arabidopsis thaliana	*Cuscuta australis*	*Solanum tuberosum*	0.206047	0.179943	0.026103	0.001004	25.989989	99.96%	*Cuscuta australis*
Arabidopsis thaliana	*Cuscuta australis*	*Solanum lycopersicum*	0.20728	0.170191	0.037088	0.000958	38.707126	99.96%	*Cuscuta australis*
Arabidopsis thaliana	*Cuscuta australis*	*Coffea canephora*	0.2209	0.185532	0.035368	0.000985	35.891519	99.96%	*Cuscuta australis*
Arabidopsis thaliana	*Cuscuta australis*	*Capsicum annuum*	0.207216	0.177334	0.029883	0.000985	30.338771	99.96%	*Cuscuta australis*
Arabidopsis thaliana	*Cuscuta australis*	*Ipomoea nil*	0.125598	0.094319	0.031279	0.000767	40.798868	99.96%	*Cuscuta australis*
Arabidopsis thaliana	*Cuscuta australis*	*Mimulus guttatus*	0.225954	0.211697	0.014257	0.001028	13.865957	99.96%	*Cuscuta australis*

The branch length was calculated using *Arabidopsis thaliana* as the outgroup. Z-statistic was used to test whether the distances between ingroups (bA, bB) to the outgroup is significantly different from 0 or not. Delta is the absolute difference between bA and bB. Z-statistics (Z) is delta/standard error (s.e.). CP (confident probability) is equal to 1 - (p value).

Supplementary Table 6. Relative rate test of *Cuscuta australis* and related species

The chi-square test was based on 1 degree of freedom.

Outgroup	Ingroup 1	Ingroup 2	Identical	Ingroup 1 specific	Ingroup 2 specific	Chi-score	p-value	Faster
Arabidopsis thaliana	*Cuscuta australis*	*Solanum tuberosum*	467586	100123	88818	676.42	<0.00001	*Cuscuta australis*
Arabidopsis thaliana	*Cuscuta australis*	*Solanum lycopersicum*	504389	107675	90153	1551.96	<0.00001	*Cuscuta australis*
Arabidopsis thaliana	*Cuscuta australis*	*Coffea canephora*	498947	113425	96542	1357.53	<0.00001	*Cuscuta australis*
Arabidopsis thaliana	*Cuscuta australis*	*Capsicum annuum*	484351	103773	90260	941.08	<0.00001	*Cuscuta australis*
Arabidopsis thaliana	*Cuscuta australis*	*Ipomoea nil*	537711	69320	54480	1778.88	<0.00001	*Cuscuta australis*
Arabidopsis thaliana	*Cuscuta australis*	*Mimulus guttatus*	485175	113596	106831	207.62	<0.00001	*Cuscuta australis*

Supplementary Table 7. Statistics of different patterns of depths of *Cuscuta australis* and *Ipomoea nil* syntenic blocks covering all *Coffea canephora* syntenic orthologs

Cuscuta australis:*Ipomoea nil*: *Coffea canephora*	Count
3:3:1	4943
2:3:1	4604
2:2:1	1981
1:2:1	1931
0:0:1	1740
1:1:1	1513
1:3:1	1037
0:1:1	1007
3:4:1	668
2:4:1	557
3:2:1	490
0:2:1	390
1:0:1	330
2:1:1	316
0:3:1	174
2:5:1	104
1:4:1	66
2:0:1	39
4:4:1	28
4:3:1	19
3:5:1	16
3:1:1	13
1:5:1	3

Note: For this statistic, every syntenic block was anchored to the chromosomes of *Coffea canephora*, and the numbers of the syntenic blocks from *Cuscuta australis* and *Ipomoea nil* covering each individual *Coffea canephora* syntenic gene were determined to obtain the syntenic coverage information, which is described by "number of syntenic block of *Cuscuta australis*:number of syntenic block of *Ipomoea nil*:1" for each *Coffea canephora* gene locus. The syntenic *Coffea canephora* genes with a specific coverage pattern were counted and plotted.

Supplementary Table 8. Results of BUSCO analysis for the genomes of *Cuscuta australis*, *Utricularia gibba*, and 7Ref-Species

	Complete BUSCOs	Complete and single-copy BUSCOs	Complete and duplicated BUSCOs	Fragmented BUSCOs	Missing BUSCOs	Total BUSCO groups searched
Arabidopsis thaliana	98.20%	97.40%	0.80%	0.40%	1.40%	1440
Mimulus guttatus	91.50%	87.40%	4.10%	3.10%	5.40%	1440
Utricularia gibba	82.60%	77.80%	4.80%	3.70%	13.70%	1440
Coffea canephora	93.80%	91.70%	2.10%	1.90%	4.30%	1440
Capsicum annuum	88.40%	85.80%	2.60%	3.10%	8.50%	1440
Solanum lycopersicum	96.40%	94.60%	1.80%	0.60%	3.00%	1440
Solanum tuberosum	94.70%	92.20%	2.50%	0.80%	4.50%	1440
Ipomoea nil	93.90%	87.10%	6.80%	1.70%	4.40%	1440
Cuscuta australis	80.60%	78.50%	2.10%	3.10%	16.30%	1440

Supplementary Table 9. Mapping ratios of RNA-Seq reads from *Cuscuta australis* and *Cuscuta pentagona* to the PacBio assembly of *C. australis*

Sample Name	Species Name	Sample ID	Hosts	Contamination Percentage	Alignment Ratio
**C10_Seeds**	*Cuscuta australis*	C10	-	-	96.06%
**C1_Bud on soybean host**	*Cuscuta australis*	C1	Soybean	0.34%	96.40%
**C3_Ovary on soybean host**	*Cuscuta australis*	C3	Soybean	0.34%	96.87%
**C4_Germinating seeds**	*Cuscuta australis*	C4	-	-	95.28%
**C5_Haustoria on tomato host**	*Cuscuta australis*	C5	Tomato	0.44%	96.75%
**C6_Prehaustoria on tomato host**	*Cuscuta australis*	C6	Tomato	3.35%	96.39%
**C7_Curling stems on tomato host**	*Cuscuta australis*	C7	Tomato	0.13%	96.87%
**C8_Stem tip on tomato host**	*Cuscuta australis*	C8	Tomato	1.21%	96.83%
**flowers_tobacco***	*Cuscuta pentagona*	flowers_tobacco.rep1	Tobacco	2.36%	78.38%
		flowers_tobacco.rep2	Tobacco	4.53%	79.30%
		flowers_tobacco.rep3	Tobacco	2.65%	80.55%
		flowers_tobacco.rep4	Tobacco	1.81%	77.39%
**flowers_tomato***	*Cuscuta pentagona*	flowers_tomato.rep1	Tomato	3.08%	79.93%
		flowers_tomato.rep2	Tomato	9.91%	75.31%
		flowers_tomato.rep3	Tomato	3.51%	78.42%
		flowers_tomato.rep4	Tomato	9.94%	75.32%
**haustorial_stems_tobacco***	*Cuscuta pentagona*	haustorial_stems_tobacco.rep1	Tobacco	3.45%	61.17%
		haustorial_stems_tobacco.rep2	Tobacco	4.33%	61.09%
		haustorial_stems_tobacco.rep3	Tobacco	2.22%	68.85%
		haustorial_stems_tobacco.rep4	Tobacco	3.04%	68.86%
**haustorial_stems_tomato***	*Cuscuta pentagona*	haustorial_stems_tomato.rep1	Tomato	1.60%	63.80%
		haustorial_stems_tomato.rep2	Tomato	2.29%	71.41%
		haustorial_stems_tomato.rep3	Tomato	2.95%	64.19%
		haustorial_stems_tomato.rep4	Tomato	2.13%	71.30%

**prehaustorial_stems_tobacco***	*Cuscuta pentagona*	prehaustorial_stems_tobacco.rep1	Tobacco	4.94%	78.25%
		prehaustorial_stems_tobacco.rep2	Tobacco	2.77%	79.56%
		prehaustorial_stems_tobacco.rep3	Tobacco	2.59%	80.34%
		prehaustorial_stems_tobacco.rep4	Tobacco	2.94%	76.26%
**prehaustorial_stems_tomato***	*Cuscuta pentagona*	prehaustorial_stems_tomato.rep1	Tomato	2.20%	72.39%
		prehaustorial_stems_tomato.rep2	Tomato	3.09%	76.03%
		prehaustorial_stems_tomato.rep3	Tomato	2.06%	74.68%
		prehaustorial_stems_tomato.rep4	Tomato	1.54%	77.65%
**seedlings***	*Cuscuta pentagona*	seedlings.rep1	-		80.23%
		seedlings.rep2	-		79.47%
		seedlings.rep3	-		80.97%
		seedlings.rep4	-		79.88%
**seeds***	*Cuscuta pentagona*	seeds.rep1	-		72.01%
		seeds.rep2	-		71.94%
		seeds.rep3	-		67.84%
		seeds.rep4	-		71.80%
**stems_tobacco***	*Cuscuta pentagona*	stems_tobacco.rep1	Tobacco	6.10%	80.70%
		stems_tobacco.rep2	Tobacco	1.94%	80.75%
		stems_tobacco.rep3	Tobacco	6.13%	80.76%
		stems_tobacco.rep4	Tobacco	4.88%	75.67%
**stems_tomato***	*Cuscuta pentagona*	stems_tomato.rep1	Tomato	4.33%	79.29%
		stems_tomato.rep2	Tomato	4.07%	80.58%
		stems_tomato.rep3	Tomato	3.71%	80.08%
		stems_tomato.rep4	Tomato	3.91%	80.51%

*RNA-Seq data of *Cuscuta pentagon* was obtained from Reference [2]. Note: Reads of host mRNAs have been filtered out before mapping was done.

Supplementary Notes

Supplementary Note 1.

Exclusion of organelle-derived contigs. We excluded the organelle-derived sequences based on the read mapping depths and the characteristic genes and structures of organellar genomes. Firstly, the Illumina pair-end reads were mapped to the PacBio genome assembly for calculating the sequencing depths in each contigs, and the 60 contigs with more than 100 × depths were selected. The previously reported plastidial and/or mitochondrial genomes of *Cuscuta gronovii*, *Cuscuta reflexa*, *Ipomonea nil*, and *Arabidopsis thaliana* (accession Nos. NC_009765.1, NC_009766.1, NC_031159.1, NC_031158.1, KX551970.1, Y08501.2 respectively) were retrieved and used as queries to search these 60 contigs using blastn[3] (v2.4.0) and tblastx[3] (v2.4.0) to mark the coding regions, and the RepBase database was employed to mark noncoding and repetitive regions using Repeatmasker[4] (v4.0.6). The contigs with the characteristic genes and genome structures of plastids and mitochondria were manually inspected and removed from the PacBio assembly.

In total, 30 contigs (Supplementary Table 2) comprising 1,906,600 bp were identified as organellar sequences, each with sequencing depths ranging from 582.3 to 3267.5 ×, and one contig C141C with a length of 85,214 bp shows the characteristics of a complete circular plastid genome (Supplementary Fig. 8).

Supplementary Note 2.

Illumina reads cleanup and assembly. *de novo* assembling the Illumina short reads was performed based on the previously reported pipeline Phusion-meta (v2.3) with several minor modifications[5]. Briefly, after removing low-quality reads containing ten or more

unique *k*-mers, the paired-end reads were clustered into thousands of groups by Phusion2[6] (v2.3) with *k*-mer at 51 bp. The reads within each cluster were assembled into contigs with N50 of 17 kb using the Fermi[7] (v1.1) and SOAPdenovo[8] (v2.04) software. All the obtained contigs from above were merged by the aligner GAP5[9] (v2.0.0b11), which were then used to build the scaffolds with the mate-paired reads from the 2-k and 5-k libraries hierarchically and iteratively with SOAPdenovo[8] (*k*-mer at 81 bp), resulting in an assembly with the N50 of 214 kb and length of 266.9 Mb.

Supplementary Note 3.

Gene anntation

1) **Identification of repetitive elements.** To identify repetitive elements in *Cuscuta australis*, we first used *de novo* prediction to build a custom database of repeats in *Cuscuta australis* with RepeatModeler[4] (v1.0.4). Then we used Repeatmasker[4] (v4.0.6) to annotate the repetitive regions in *Cuscuta australis* genome based on our custom database and the models of repeats from RepBase[10] (release 22.10). TRF[11] (v4.0.7b) was used to improve finding of simple tandem repeats. After the redundant results were removed, the repetitive elements were annotated using Repeatmasker (Supplementary Table 4). In this manner, 58% of the genome regions were identified as the repetitive regions. To facilitate comparison of the repeats between *Cuscuta australis* and *Ipomoea nil*, the same pipeline was also used for the genome of *Ipomoea nil*. A total of 2138 and 2143 families of repetitive elements were identified in *Cuscuta australis* and *Ipomoea nil*, respectively. To compare the evolutionary dynamics of LTRs, the phylogenetic trees for two largest families, *LTR/Copia* and *LTR/Gypsy*, were reconstructed

(Supplementary Fig. 1). Sequences of *LTR/Copia* and *LTR/Gypsy* were first aligned using famsa[12] (v1.2.1), and the phylogenetic trees were then reconstructed using Fasttree[13] (v2.1.9).

2) **Gene model prediction.** Three different methods were employed for gene model prediction:

1. Homology-based prediction

Protein sequences of 7Ref-Species (*Arabidopsis thaliana*, *Ipomoea nil*, *Solanum tuberosum*, *Solanum lycopersicum*, *Capsicum annuum*, *Coffea canephora*, and *Mimulus guttatus*) were first aligned to the masked genome of *Cuscuta australis* using tblastn[3] (v2.4.0) with e-value < 1e-5 to find corresponding homologous regions in *Cuscuta australis*. Genewise[14] (v2.0) was then utilized to generate accurate gene structures in these regions using the protein sequences of 7Ref-Species as the references.

2. Transcriptome-based prediction

RNA-seq data from different tissues of *Cuscuta australis* were aligned to the masked genome using TopHat[15] (v2.0.8). Cufflinks[16] (v2.1.1) was then used to construct genes. To find genes supported by *Cuscuta pentagona* transcript evidence, we assembled transcripts of *Cuscuta pentagona* and aligned against the genome of *Cuscuta australis* using blat[17] (v. 35).

3. *De novo* gene prediction

Gene models of *Cuscuta australis* were first predicted using *de novo* prediction methods Augustus[18] (v2.5.5), GlimmerHMM[19] (v3.0.1), GeneScan[20]

(v1.0), SNAP[21] (version 2006-07-28), and Geneid[22] (v1.4) . Training set for *de novo* prediction included the complete gene model information from 7Ref-Species and the transcriptome data obtained from *Cuscuta australis* and *Cuscuta pentagona* (assembled using Trinity[23] (r20140717) and structurally annotated with PASA[24] (v2.0.2)).

De novo prediction, homology-based prediction, and transcriptomic evidence were further integrated using EVM[25] (v1.1.1), resulting in a non-redundant complete gene-set. UTR regions were identified by integrating transcript evidence with gene annotation results using PASA[24] (v2.0.2).

3) **Pseudogene identification.** Given that some of pseudogenes may be mistakenly identified as retrotransposons, we firstly remasked the genome with a lowered standard: Retrotranspons and DNA transposons were identified with RepeatMasker[4] (v4.0.6) based on the RepBase[10] database (release 22.10), other than *de novo* prediction as previously described. Thereafter, low-complexity repeats were identified using Tandem Repeats Finder[11] (v4.0.7b). The interspersed repeats were hard-masked (masked as N). Considering some proteins also contain tandem repeats, low complexity repeats were soft-masked (masked as lowercase) so that a blast hit can extend from a seed hit to the low-complexity repetitive regions.

The procedure of pseudogene identification was adopted from Zou et al[26]. To identify the locations of the homologs of all the gene models of 7Ref-Species in *Cuscuta australis* genome, all protein sequences from the 7Ref-Species genomes were used as queries to search against the remasked genome of *Cuscuta australis*

using tblastn[3] (v2.4.0). A total of 18,823 homologous regions were found. After removing those overlapping with those previously identified gene loci, structures of genes missed in the previous annotation were predicted using GeneWise[14] (v2.0), which built fine alignments between query protein sequences and the homologous regions in *Cuscuta australis*. Predicted gene structures with mutations leading to frame-shifts and premature stop codons were selected as candidate pseudogenes. As some of the query sequences might be mistakenly annotated in structures, false positives of pseudogenes could be identified. To minimize this possibility, we manually examined if the mutations could be supported by most of the query sequences in multiple sequence alignments. Genes passed the manual inspections were considered to be pseudogenes. Newly annotated genes without evidence of being pseudogenes were added to the previously annotated gene-set to improve the completeness for gene loss analysis later. To this end, we obtained 19,805 protein-coding genes and 1,283 pseudogenes.

4) **Functional annotation.** The annotated protein sequences were searched against KEGG (release 53), NR (version 20150810), and Swiss-Prot (version 20150821) using blastp[3] (v2.4.0) (e-value < 1e-5), and the best hits were retained as putative function annotations. InterproScan (v4.8) were used to identify domain information in *Cuscuta australis* genes. In total, 90.2% of all the genes were functionally annotated.

Supplementary Note 4.

Phylogeny and divergence times.

Phylogenetic tree was build using RAxML[27] (v8.2.11) with the protein sequences of single-copy orthogroups (Fig. 1b). A long branch was detected in *Cuscuta australis*, suggesting a rapid evolution rate. This was further validated using two cluster analysis and Tajima's relative rate test[28-30] (Supplementary Table 5 and Supplementary Table 6). Divergence time was estimated using the mcmctree (burn-in=10,000, sample-number=100,000, sample-frequency=2) embedded in the PAML package[31] (v4.9e) with calibration set to 106.8-124.8 Mya between *Arabidopsis thaliana* and *Mimulus guttatus* and 6.5-7.4 Mya between *Solanum lycopersicum* and *Solanum tuberosum* (Supplementary Fig. 2). These divergence times were obtained from timetree (http://www.timetree.org).

Supplementary Note 5.

Expansion and contraction of *Cuscuta australis* gene families.

The protein sequences from *Cuscuta australis*, *Utricularia gibba*, and the 7Ref-Species were firstly all-vs-all aligned using blastp[3] (v2.4.0). E-value of each hit was transformed into *W* index [1] ranging from 0 to 100 using the following formula.

$$W = Min(100, \mathrm{ROUND}(\frac{-\log_{10}(Evalue)}{2}))$$

Thereafter, *W* indices were passed to hcluster[1] (v0.5.1) as weight matrices to build gene families with the parameters “-m 170 -w 0 -s 0.34 -O”. A total of 13981 gene families were constructed using this method, covering 92% of the total. To directly compare sizes of gene families between species, we computed the *F*-indices, which describe the size differences of conserved gene families in 7Ref-Species (conserved gene families must

exist in *Arabidopsis* and at least five out of the six remaining species in the 7Ref-Species), using the formula below.

In this formula, i refers to a given species and j refers to a given family, c_{ij} is the number of genes in j family in i species, and N_j is the total number of genes in j family of all species, and S represents the number of species. F-index is a modified log2-fold-change coefficient ($\log_2 \frac{c_{ij}}{N_j}$), so the relationship between F-index and $\log_2 \frac{c_{ij}}{N_j}$ is linear. F-indices range from 0 to 1, and when F_{ij} = 0.5, in species i, the gene number in gene family j equals to the average size of this gene family in all species; if F_{ij} = 0, there are no genes in family j in species i, and if F_{ij} = 1, it indicates that only species i, but not the others, harbors the gene family j. Thereafter, a box plot of F-indices of the families (data are listed in Supplementary Data 1a), that are conserved in 7Ref-Species, for all species was constructed (Fig. 2b).

$$F_{ij} = \frac{\log_2\left(a\frac{c_{ij}}{N_j} + \frac{1}{2}\right) + 1}{\log_2\left(a + \frac{1}{2}\right) + 1}$$

$$a = \frac{S^2 - 2S}{2}$$

To investigate expansions and contractions of gene families, we used the gain-and-death (GD) model in BadiRates[32] (v1.35), a program that estimates family turnover rates by likelihood-based methods. Species tree and gene family number in each species were used as the input file for BadiRates[32]. To calculate which lineages were significantly expanded or contracted, we used the free model in BadiRates to estimate the sizes of the ancestral gene families in all clades of the species tree. For the branches whose gene

families did not experience family size changes, they were set to be the background branches, which have the same family turnover rate; thereafter, based on this model, we re-estimated the likelihood, and the model was regarded as the null hypothesis. For branches that experienced size changes, an alternative hypothesis for each branch was built by forcing the given branch to follow the same turnover rate with the background branches. A branch that experienced size changes was considered to be significant, if AIC (alternative hypothesis) - AIC (null hypothesis) > 2 (Akaike's information criterion (AIC)[33] was computed from the likelihood and numbers of parameters in each model). Hereafter, the significantly expanded and contracted gene families in all eight species were obtained (details are in Supplementary Data 1a).

Supplementary Note 6.

Identification of gene loss in the *Cuscuta australis* and *Utricularia gibba* genome

We developed a pipeline to identify the orthologous gene clusters in the genomes of *Cuscuta australis, Utricularia gibba*, and the 7Ref-Species (Supplementary Fig. 6), which considers the phylogenetic and the collinear information of genes. In order to obtain a rather complete overview of the lost genes, pseudogenes in *Cuscuta australis* and *Utricularia gibba* were also integrated into the gene models, by masking the frame-shift and premature stop codon mutation sites to X and N on the amino acid and nucleotide level, respectively. Considering the possibility that genes with frame-shifts and premature stop codons identified *in silico* may still be translated to proteins which retain their functions, these genes were treated as normal genes and input into the gene loss analysis.

The details of the pipeline procedure are summarized as the following:

1. BUSCO analysis

The genome sequences of *Cuscuta australis*, *Utricularia gibba*, and 7Ref-Species were input into the BUSCO (v3) pipeline[34], and the *Solanum lycopersicum* gene model was used as the training set for Augustus. The results are presented in Supplementary Table 8.

2. Identification of the orthogroups from phylogenetic trees

All the predicted gene models in the nine species were clustered using orthoMCL[35] (v2.0.9) with the parameters set as percentMatchCutoff=50, evalueExponentCutoff=-5, inflation=1.5, resulting in 25,330 OrthoMCL groups; phylogenetic analysis was carried out for each group obtained above using protein alignments produced by t-Coffee[36] (v11.00) (aligner set: mafftgins_msa, muscle_msa, kalign_msa, and t_coffee_msa), and the codon alignments were deduced from protein alignments. To further optimize the tree topologies, we used TreeBeST[1] (v1.9.2) to construct five different types of phylogenetic trees: two maximum likelihood (ML) trees based on protein alignments and codon alignments, respectively, and three neighbour-joining (NJ) trees based on codon alignments with p-distances, dN distances, and dS distances. The final trees were generated by the TreeBeST-implemented tree merging algorithm, which integrates species tree and the five trees, considering the advantages of DNA- and protein-based trees, bootstrap values, and the minimization of gene duplication/loss numbers.

Arabidopsis is the outgroup for *Cuscuta*, *Utricularia*, and the other six autotrophic reference species belong to the lamiids; thus, all the genes in each orthogroup must have the same *Arabidopsis* ortholog; thus, these genes in a specific orthogroups should closely cluster with the *Arabidopsis* outgroup ortholog gene in a branch of the gene family phylogenetic tree. Since *Arabidopsis* is the most remotely related with the other species

and has been intensively studied for gene functions, tree topologies can be constructed unambiguously and the functions of orthogroups can be assigned accordingly. To divide each phylogenetic tree into individual orthogroups, we firstly chose the clades containing *Arabidopsis* outgroups and then treated the remaining clades with the same levels as those containing *Arabidopsis* outgroups as independent orthogroups. A total of 21,487 orthogroups (≥ 2 species) were identified using this method, among which, 11,995 are conserved in the 7Ref-Species (considering that there could be mistakes in the genome annotation of 7-Ref species, a "conserved orthogroup" is formed only if it contains the orthologs from *Arabidopsis* and all or five out of the remaining six species from 7Ref-Species), including 16,799 *Arabidopsis* genes (Supplementary Data 1b and 1c). Among the conserved orthogroups, 1869 and 3236 orthogroups in *Cuscuta australis* and *Utricularia gibba*, respectively, were identified to potentially have gene losses (Supplementary Data 1b). These orthogroups are named as orthogroups with potential gene losses (OPGL).

Various factors could affect gene loss identification, including OrthoMCL grouping, phylogenetic reconstruction of gene trees, and genome masking and incomplete transcriptome information during the annotation step. To minimize false positives that could be caused by these factors, the previously identified gene losses in both *Cuscuta australis* and *Utricularia gibba* were further inspected by the following pipeline (Supplemental Fig. 6):

1) tblastn (E-value threshold = 1e-10) search was first applied to search the unmasked *Cuscuta australis* genome and the assembled unmapped *Cuscuta australis* transcriptome (See methods "Transcriptome analysis" for detail) with all the genes

in the 1869 orthogroups, which appeared to have lost *Cuscuta australis* members. After tblastn search of the *Cuscuta australis* genome, the resulted hits were concatenated (because of possible introns), if the distances of the adjacent matches were within 10 kb. In this manner, we could find all the homologs of the genes in all OPGLs in *Cuscuta australis*, if there were any. We performed the same analysis for *Utricularia gibba.*

2) All the homologs obtained from the Step a. were aligned against the annotated genes in the respective *Cuscuta australis* or *Utricularia gibba* genome. If a homolog had been annotated (covering at least 80%), it was regarded as successfully annotated; otherwise, the homolog was assigned as unannotated.

3) The sequences of all the newly identified unannotated homologs were extended to include 5 kb of both upstream and downstream border sequences. Genewise[14] (v2.2.0) was exploited to annotate the gene models of these homologs, and the query sequences used in tblastn search were used as the Genewise references. The output protein sequences from Genewise annotation must cover at least 50% of the reference sequences. Since multiple reference sequences from different genomes were used as queries, redundant output protein sequences exist. The redundant sequences were removed with CD-HIT[37] (v4.6) (identity threshold 90%), leaving only the longest sequences.

4) All the sequences obtained from Step c. and the gene models from 7-Ref species and the previously annotated *Cuscuta australis* and *Utricularia gibba* were integrated into a database. All the sequences in each OPGL were used as queries to perform blastp[3] (v2.4.0) (E-value threshold 1e-5) against this database. The resulted subjects

from each query in a given OPGL were scored by transforming the E-value of each subject into *W* index (See Supplementary Note 2. "Construction of gene families"). After all the genes in a given OPGL are done with blastp as queries, the scores of the same subjects were summed to obtain their final scores. Then, the subjects identical to those in the OPGL were removed from further analysis. The remaining subjects were subsequently classified according their species, and the top five subjects with the highest scores were acquired for each species.

5) These subjects and the sequences in the respective OPGL were used for phylogenetic analysis (clustalW and Fasttree; reliable clades must have bootstrap values greater than 80%). If certain genes from *Cuscuta australis* or *Utricularia gibba* appeared in the clade of OPGL, these orthogroups are no longer considered to be involved in gene loss.

A total of 1494 and 1555 orthogroups were still identified to have gene loss in *Cuscuta australis* and *Utricularia gibba*, respectively.

3. Identification of orthogroups from collinear fragment information

Although the method described in "1. Identification of the orthogroups from phylogenetic trees" can generate almost complete information of orthogroups using protein sequences, it ignores the synteny information among different species. Thus, a pipeline was developed to identify orthogroups based on syntenic gene pairs (Supplementary Fig. 6), in order to complement the orthogroups obtained from the above step.

Firstly, protein sequences from *Cuscuta australis*, *Arabidopsis thaliana*, *Coffea canephora*, and *Ipomoea nil* were all-vs-all aligned using blastp[3] (v2.4.0) with the E-value set to 1e-5. Then the results from blastp and the information of gene loci in all

Cuscuta australis, *Arabidopsis thaliana*, *Coffea canephora*, and *Ipomoea nil* were used to feed MCScanX[38] (version 201610) to obtain the information about the collinear fragments between all pairs of these four species.

These fragments were originated from two processes, namely, genome duplication (including the gamma triplication event, before the speciation between lamiids and Arabidopsis) and the divergence between *Cuscuta australis*, *Arabidopsis thaliana*, *Coffea canephora*, and *Ipomoea nil*. To construct orthogroups, we utilized the previously established orthogroups (from “1) Identification of the orthogroups from phylogenetic trees”) to identify a set of collinear fragments derived from speciation (MCScanX_h (version 201610)). These fragments were further used as a reference to guide the selection of MCScanX results, resulting in fragments that are suitable for identification of orthology (the noise of paralogs generated by genome duplication could be removed).

In this manner, 54,165 genes among the 116,404 genes in *Cuscuta australis*, *Arabidopsis thaliana*, *Coffea canephora*, and *Ipomoea nil* were clustered into 14,978 orthogroups. Using these orthogroups based on collinearity, the orthogroups obtained from phylogenetic analysis were corrected: 93 out of 1495 orthogroups, which were formerly considered to have no *Cuscuta australis* members, could retrieve their *Cuscuta australis* orthologs.

Functions of all conserved orthogroups were assigned based on the *Arabidopsis* orthologs (Supplementary Data 1b and 1c), and GO enrichment of the these 1402 orthogroups whose *Cuscuta australis* members are absent indicated that “transport”, “photosynthetic”, “regulation of transcription”, and “response to stimulus” were overrepresented (Supplementary Data 2a, Supplementary Data 2b).

4. Manual inspection on the lost genes of interest.

To manually inspect the lost genes in *Cuscuta australis*, which are mentioned in the main text and whose orthologs in *Arabidopsis* play important roles in leaf and root development, nutrient transport, flowering, and defenses, etc. (Supplementary Data 1c), the following procedure was adopted:

1) Inspection of the PacBio assembly

a Exonerate[39] (v2.2.0) software was used (--model protein2genome --percent 50) to map the protein sequences of *Arabidopsis* orthologs to *Cuscuta australis* PacBio assembly, in order to ensure the target gene loci have been annotated as functional or pseudogenes.

b Each *Arabidopsis* protein sequence was set as a query to perform blastp[3] (v2.4.0) against the gene models of *Cuscuta australis* and 7Ref-Species (E-value=1e-5). The obtained subject sequences were used to conduct multiple-sequence alignment (MSA) with the MUSCLE[40] (v3.8.31) software (in case of too many subject sequences, i.e. over 100, MCL (inflation=1.4) was used to cluster the subject sequences into smaller groups, and the group containing the query was used for MSA). The sequences with poor alignment quality in MSA were manually removed to redo MSA; this procedure was repeated until no sequences were considered to have poor alignment quality. Fasttree[13] (v2.1.9) was adopted to construct maximum-likelihood trees, and based on the tree topologies, whether there were gene losses was confirmed.

2) Inspection in the Illumina assembly

To minimize the possibility of sequencing and assembly errors during PacBio data analysis, which led to false positives of gene loss, we also inspected the gene loss data in the Illumina assembly.

a Exonerate[39] (v2.2.0) software was used (--model protein2genome --percent 50) to map the protein sequences of *Arabidopsis* orthologs to *Cuscuta australis* Illumina assembly, and the target loci were annotated with Genewise[14] (v2.2.0) to obtain protein sequences.

b Each *Arabidopsis* protein sequence was set as a query to perform blastp[3] (v2.4.0) against the gene models of all the seven autotrophic species (E-value=1e-5). The subject sequences and the Genewise-annotated *Cuscuta australis* protein sequences were analyzed through the pipeline described in 1b) of this Section.

Supplementary Note 7.

Whole genome duplication analysis

Coffea canephora is a plant that never underwent WGD after the ancient gamma event. To gain insight into the evolution of *Cuscuta australis* genome after the gamma event, the orthologous pairs of *Cuscuta australis*, *Ipomoea nil*, and *Coffea canephora* genes in the identified orthogroups (these gene pairs were derived from speciation and/or gene duplication after the gamma event) were fed to MCScanX_h[38] (version 201610) for construction of the post-gamma-event syntenic blocks (Supplementary Fig. 6).

We next anchored every syntenic block to the chromosomes of *Coffea canephora*. There are 21969 *Coffea canephora* genes that were found to be covered by at least one syntenic block. Thereafter, we counted the numbers of the syntenic blocks from *Cuscuta australis*

and *Ipomoea nil* covering each individual *Coffea canephora* gene from these 21969 gene loci, so that we could obtain the syntenic coverage information, which is described by "number of syntenic block of *Cuscuta australis*:number of syntenic block of *Ipomoea nil*:1" for each *Coffea canephora* gene locus. Among 21969 *Coffea canephora* gene loci, 4943 (22.4%) and 4604 (21.0%) showed syntenic coverage of 3:3:1 and 2:3:1, respectively (Supplementary Fig. 3 and Supplementary Table 7), and these two types were the most common ones. These data suggest that there was a genome triplication event in both *Cuscuta* and *Ipomoea* genome after the gamma event. These two most common types of syntenic blocks were anchored to their corresponding *Coffea canephora* gene loci, and positions of these *Coffea canephora* loci were plotted over the chromosomes (Supplementary Fig. 4), and they appear to scatter relatively evenly across the whole *Coffea canephora* genome, suggesting that the genome triplication event occurred at the whole genome level rather than on certain chromosomes or specific chromosome regions (Supplementary Fig. 4). A synteny circos figure was drawn using the Circos[41] (v 0.69) tool for the biggest syntenic blocks between *Cuscuta australis*, *Ipomoea nil*, and *Coffea canephora* chromosome 2 (Fig. 1c).

To investigate whether the triplication event is shared by *Ipomoea* and *Cuscuta*, we chose 574 *Coffea canephora* genes that have at least two ortholog genes in both *Ipomoea nil* and *Cuscuta australis* among all the homologous genes in the syntenic blocks. For these 574 *Coffea canephora* genes, each gene was grouped with its orthologs, and these groups were used to construct phylogenetic trees. For these trees, we assumed two models (Supplementary Fig. 5): Model A: WGD happened before the speciation between *Ipomoea* and *Cuscuta*; Model B: WGD happed independently after the speciation

between *Ipomoea* and *Cuscuta*. If model A outperforms, orthologous gene pairs from *Cuscuta australis* and *Ipomoea nil*, respectively, would be grouped together; otherwise, paralogous genes of *Cuscuta australis* and *Ipomoea nil* would be grouped separately. We tested those two models by searching the 574 phylogenetic trees for clades that fit into different models (bootstrap > 0.7). Only 36 clades supported Model B, while Model A was supported by 1003 clades.

Considering the results from phylogenetic and syntenic analyses, we concluded that a common triplication event took place before the speciation between *Cuscuta* and *Ipomoea*.

Supplementary Note 8.

Transcriptome analysis

1. Transcriptome assembly and expression level analysis

Reads from *Cuscuta australis* transcriptomes and *Cuscuta pentagona* transcriptome [2] were first aligned against the corresponding host genome sequences using bowtie2[42] (v2.2.4) to remove possible host mRNA contamination. The filtered reads were aligned against *Cuscuta australis* genome independently using HISAT2[43] (v2.0.5) with parameters "-mp 3,1", achieving an average ratio of the mapped reads of 96% and 75% (Supplementary Table 9). Unmapped RNA-Seq reads of *Cuscuta australis* were extracted and de novo assembled using Trinity[23] (r20140717). These assembled transcripts were used to support gene loss analysis (see Supplementary Note 4). Transcript abundances were calculated using StringTie[44] (v1.3.0). Genes, whose average FPKM values at least 1-fold greater in prehaustoria/haustoria than in other organs, were considered to be principally expressed in prehaustoria/haustoria (Supplementary Data 3f).

The transcriptome datasets were retrieved from the Tomato Functional Genomics Database (http://ted.bti.cornell.edu/cgi-bin/TFGD/digital/sample.cgi?ID=D004), including data from tomato roots, fruits, leaves, seeds, flowers, and buds. *Ipomoea nil* transcriptome datasets (embryos, flowers, leaves, roots, seeds, and stems) were obtained from DDBJ (ftp://ftp.ddbj.nig.ac.jp/ddbj_database/dra/fastq/DRA002/DRA002647) in raw data format, and these data were processed with HISAT2 and StringTie as describe above to acquire transcript level values.

2. Identification of principally expressed tissues for all orthogroups in *Cucuta pentagona*, *Solanum lycopersicum*, and *Ipomoea nil*

A method based on Yang et al.[45] was developed to identify principally expressed tissues (PETs). Using the orthogroup information (Supplementary Data 1b) and tissue-specific transcriptomes of these three species, firstly we searched the transcriptome data for identification of the principally expressing tissues of all the genes in these orthogroups. For each orthogroup, the transcript levels of these three species' orthologs in their respective different tissues of *Cucuta pentagona*, *Solanum lycopersicum*, and *Ipomoea nil* were assigned according to their respective transcriptome data; in case of more than one gene member exist in a species in the orthogroup, the highest transcript levels were used, and the transcript levels in different tissues within a species were compared; in a given tissue, if the orthologue's transcript level is at least 1-fold greater than the average levels in all the other tissues, this tissue was assigned as the principally expressing tissue for this specific orthologue (orthologues, in case of more than one orthologue of a species exist). This was done for all the orthogroups.

Then we searched all the orthogroups for the *Cuscuta* orthologs whose principally expressing tissue is prehautoria/haustoria (1299 orthogroups were found), and the corresponding *Solanum lycopersicum* and *Ipomoea nil* orthologs' principally expressing tissues were recorded. Finally, the principally expressing tissue of these 1299 orthogroups in *Solanum lycopersicum* (505 orthogroups) and *Ipomoea nil* (515 orthogroups) was found to be root (Supplementary Data 1d).

The orthogroups whose *Cuscuta* members are missing were used to analyze the principally expressed tissues of the *Solanum lycopersicum* and *Ipomoea nil* orthologs, in order to gain insight into the expression patterns of the corresponding orthologs of the lost genes in these autotrophic plants. Finally, the principally expressing tissues of 1402 orthogroups, which do not have *Cuscuta* members (gene loss in *Cuscuta*), in *Solanum lycopersicum* and *Ipomoea nil* are listed in Supplementary Data 1d.

Supplementary Note 9.

Analysis of *R* genes, *TPS*s, *P450*s, and *RLK*s.

R (*resistance*) genes are critical for plant immunity. To discover *R* genes in *Cuscuta australis* genome, we first screened for the presence of NB-ARC domain (PF00931) with HMMER[46] (version 3.0), resulting in a total of 15 *R* genes in *Cuscuta australis*. Identification of NB-ARC domain was also done for the 7Ref-Species to identify the *R* genes in these reference species (Supplementary Data 3b).

Terpenes are important chemicals which are involved in plant defenses against insects and pathogens. Similarly, we identified a total of eight *terpene synthase* (*TPS*) genes, discovered by requiring the presence of both the N-terminal domain PF01397 and C-

terminal domain PF03936 in the *Cuscuta australis* genome. We found 9 *TPS* genes in *Cuscuta australis*, and the same method was also applied to search for *TPS*s the 7Ref-Species (Supplementary Data 3c).

P450s are monooxygenases, which are critical for metabolism, including production of various plant secondary metabolites. P450 genes were identified using PFAM with PF00067 using HMMER[46] (version 3.0). A total of 89 *Cuscuta australis* P450 genes (Supplementary Data 3d) were identified in *Cuscuta australis* genome, and this is much less than in the 7Ref-Species (Supplementary Data 3d).

Receptor-like kinases (RLKs) are one of the largest gene families in the plant kingdom, constituting of a predicted signal sequence, single transmembrane region, and cytoplasmic kinase domain[47,48]. They play important roles in plant growth, development, and stress responses[49-51]. In *Arabidopsis thaliana*, more than 600 members of RLKs have been reported[52], part of them are functionally characterized, such as *FLS2*[53] and *CLAVATA1*[54].

A published complete list of *Arabidopsis RLK*s (610 genes) were used as the queries [52] to search in the gene families obtained from gene cluster information of *Cuscuta australis* and 7Ref-Species. We identified 339 *RLK* genes in the *Cuscuta australis* genome, which is much less than in the 7Ref-Species (Supplementary Data 3e).

Supplementary Note 10.

Test of positive selection and relaxed purifying selection.

HYPHY package[55] (v2.3.7) was selected for testing positive selection[56] and relaxed purifying selection[57].

1. Testing positive selection:

For each orthogroup, clustalW[58] (v2.1) was used to achieve multiple sequence alignment for all protein sequences, and Fasttree[13] (v2.1.9) was employed to reconstruct the phylogenetic tree. The backtrans module in the TreeBeST[1] (v1.9.2) was then used to align nucleotide sequences based on protein alignments. The phylogenetic trees and nucleotide alignments were input into aBSREL[56] to test positive selection in all branches of the phylogenetic trees. The branches which were significantly (Corrected P-value <= 0.05) positively selected were acquired. For a given gene, if the positively selection was not specific to *Cuscuta* or *Utricularia*, namely, positive selection could also be found in ancestral branches, this gene was no longer considered to be positively selected. The positively selected genes were further filtered: If any of them have values of K < 1 and P < 0.05 in the test of relaxed purifying selection (see below), they were removed from the positively selected genes. The full list of positively selected genes can be found in Supplementary Data 3g.

2. Testing relaxed purifying selection

The phylogenetic trees and nucleotide alignments from above were fed to RELAX[57]. The General Descriptive model embedded in RELAX was used to estimate K values for all the branches in the phylogenetic trees. The branches with the K values smaller than 1 were considered to have potentially experienced relaxed purifying selection. Each of these branches was further treated as the foreground branch and the rest branches were set to be the backgrounds, and K and P values were recalculated. All the branches with K < 1 and P < 0.05 were considered to have experienced relaxed purifying selection. For a given gene, if the relaxed purifying selection was not specific to *Cuscuta* or *Utricularia*,

namely, relaxed purifying selection could also be found in the ancestral branches, this gene was no longer considered to have experienced relaxed purifying selection. Full list of relaxed purifying selected genes can be found in Supplementary Data 3h.

Supplementary Note 11.

Photosynthesis measurements

1. Chlorophyll fluorescence measurement

The values of electron flow through PSII (ETR(II)) was measured with an imaging fluorometer (the Imaging PAM M-Series Chlorophyll Fluorescence System, Heinz-Walz Instruments, Effeltrich, Germany) connected to a computer with the control software. ETR(II) was calculated according to Genty et al. (1989) and Schreiber et al. (1995). To develop light response curves, the plants were light-adapted for at least 20 min. *C. australis* showed very low ETR(II), and the values were much lower than those of *Nicotiana tabacum*.

2. Gas exchange measurement

All measurements of gas exchange were performed on clear days by clamping one or several stems from *Cuscuta australis* into the chamber of GFS-3000 system (Portable Gas Exchange Fluorescence System GFS-3000, Heinz-Walz Instruments, Effeltrich, Germany). For gas exchange measurements, relative humidity was maintained at approximately 60%, leaf temperatures at 25 °C and CO_2 concentration of 400 μmol mol^{-1} for *C. australis*. Light-saturated photosynthetic rates at photosynthetic photon flux density intensities form 0 to 600 μmol m^{-2} s^{-1} were measured. The light response curves at photosynthetic photon flux density intensities from 1000 to 0 μmol m^{-2} s^{-1} were also

determined. However, the photosynthetic rates of *C. australis* were so low that we could not obtain stable and reliable data after several attempts (the values are far below the detection limit).

Supplementary References

1 Vilella, A. J. *et al.* EnsemblCompara GeneTrees: Complete, duplication-aware phylogenetic trees in vertebrates. *Genome Research* **19**, 327-335, doi:10.1101/gr.073585.107 (2009).

2 Ranjan, A. *et al.* De novo assembly and characterization of the transcriptome of the parasitic weed dodder identifies genes associated with plant parasitism. *Plant Physiol* **166**, 1186-1199, doi:10.1104/pp.113.234864 (2014).

3 Altschul, S. F., Gish, W., Miller, W., Myers, E. W. & Lipman, D. J. Basic Local Alignment Search Tool. *J Mol Biol* **215**, 403-410, doi:DOI 10.1006/jmbi.1990.9999 (1990).

4 Smit, A., Hubley, R & Green, P. *RepeatMasker Open-4.0*, <http://www.repeatmasker.org> (2013-2015).

5 Peng, Z. *et al.* The draft genome of the fast-growing non-timber forest species moso bamboo (Phyllostachys heterocycla). *Nat Genet* **45**, 456-461, 461e451-452, doi:10.1038/ng.2569 (2013).

6 Mullikin, J. C. & Ning, Z. The phusion assembler. *Genome Res* **13**, 81-90, doi:10.1101/gr.731003 (2003).

7 Li, H. FermiKit: assembly-based variant calling for Illumina resequencing data. *Bioinformatics* **31**, 3694-3696, doi:10.1093/bioinformatics/btv440 (2015).

8 Luo, R. *et al.* SOAPdenovo2: an empirically improved memory-efficient short-read de novo assembler. *Gigascience* **1**, 18, doi:10.1186/2047-217X-1-18 (2012).

9 Bonfield, J. K. & Whitwham, A. Gap5--editing the billion fragment sequence assembly. *Bioinformatics* **26**, 1699-1703, doi:10.1093/bioinformatics/btq268 (2010).

10 Bao, W. D., Kojima, K. K. & Kohany, O. Repbase Update, a database of repetitive elements in eukaryotic genomes. *Mobile DNA-Uk* **6**, doi:10.1186/s13100-015-0041-9 (2015).

11 Benson, G. Tandem repeats finder: a program to analyze DNA sequences. *Nucleic Acids Research* **27**, 573-580, doi:DOI 10.1093/nar/27.2.573 (1999).

12 Deorowicz, S., Debudaj-Grabysz, A. & Gudys, A. FAMSA: Fast and accurate multiple sequence alignment of huge protein families. *Sci Rep* **6**, 33964, doi:10.1038/srep33964 (2016).

13 Price, M. N., Dehal, P. S. & Arkin, A. P. FastTree 2-Approximately Maximum-Likelihood Trees for Large Alignments. *Plos One* **5**, doi:10.1371/journal.pone.0009490 (2010).

14 Birney, E., Clamp, M. & Durbin, R. GeneWise and Genomewise. *Genome Res* **14**, 988-995, doi:10.1101/gr.1865504 (2004).

15 Trapnell, C., Pachter, L. & Salzberg, S. L. TopHat: discovering splice junctions with RNA-Seq. *Bioinformatics* **25**, 1105-1111, doi:10.1093/bioinformatics/btp120 (2009).

16 Trapnell, C. *et al.* Differential gene and transcript expression analysis of RNA-seq experiments with TopHat and Cufflinks. *Nat Protoc* **7**, 562-578, doi:10.1038/nprot.2012.016 (2012).

17 Kent, W. J. BLAT - The BLAST-like alignment tool. *Genome Research* **12**, 656-664, doi:10.1101/gr.229202 (2002).

18 Stanke, M., Steinkamp, R., Waack, S. & Morgenstern, B. AUGUSTUS: a web server for gene finding in eukaryotes. *Nucleic Acids Research* **32**, W309-W312, doi:10.1093/nar/gkh379 (2004).
19 Majoros, W. H., Pertea, M. & Salzberg, S. L. TigrScan and GlimmerHMM: two open source ab initio eukaryotic gene-finders. *Bioinformatics* **20**, 2878-2879, doi:10.1093/bioinformatics/bth315 (2004).
20 Burge, C. & Karlin, S. Prediction of complete gene structures in human genomic DNA. *J Mol Biol* **268**, 78-94, doi:DOI 10.1006/jmbi.1997.0951 (1997).
21 Leskovec, J. & Sosic, R. SNAP: A General-Purpose Network Analysis and Graph-Mining Library. *Acm T Intel Syst Tec* **8**, doi:10.1145/2898361 (2016).
22 Parra, G., Blanco, E. & Guigo, R. GeneID in Drosophila. *Genome Research* **10**, 511-515, doi:DOI 10.1101/gr.10.4.511 (2000).
23 Grabherr, M. G. *et al.* Full-length transcriptome assembly from RNA-Seq data without a reference genome. *Nature Biotechnology* **29**, 644, doi:10.1038/nbt.1883 (2011).
24 Haas, B. J. *et al.* Improving the Arabidopsis genome annotation using maximal transcript alignment assemblies. *Nucleic Acids Research* **31**, 5654-5666, doi:10.1093/nar/gkg770 (2003).
25 Haas, B. J. *et al.* Automated eukaryotic gene structure annotation using EVidenceModeler and the program to assemble spliced alignments. *Genome Biol* **9**, doi:10.1186/gb-2008-9-1-r7 (2008).
26 Zou, C. *et al.* Evolutionary and expression signatures of pseudogenes in Arabidopsis and rice. *Plant Physiol* **151**, 3-15, doi:10.1104/pp.109.140632 (2009).
27 Stamatakis, A. RAxML version 8: a tool for phylogenetic analysis and post-analysis of large phylogenies. *Bioinformatics* **30**, 1312-1313, doi:10.1093/bioinformatics/btu033 (2014).
28 Tajima, F. Statistical-Method for Testing the Neutral Mutation Hypothesis by DNA Polymorphism. *Genetics* **123**, 585-595 (1989).
29 Nei, M. K., S. *Molecular evolution and phylogenetics*. (Oxford University Press, 2000).
30 Takezaki, N., Rzhetsky, A. & Nei, M. Phylogenetic Test of the Molecular Clock and Linearized Trees. *Molecular Biology and Evolution* **12**, 823-833 (1995).
31 Yang, Z. H. PAML 4: Phylogenetic analysis by maximum likelihood. *Molecular Biology and Evolution* **24**, 1586-1591, doi:10.1093/molbev/msm088 (2007).
32 Librado, P., Vieira, F. G. & Rozas, J. BadiRate: estimating family turnover rates by likelihood-based methods. *Bioinformatics* **28**, 279-281, doi:10.1093/bioinformatics/btr623 (2012).
33 Burnham, K. P. & Anderson, D. R. *Model Selection and Multi-Model Inference: A Practical Information-Theoretic Approach*. (Springer, 2002).
34 Waterhouse, R. M. *et al.* BUSCO applications from quality assessments to gene prediction and phylogenomics. *Genome Biol Evol* **35**, 543-548, doi:10.1093/molbev/msx319 (2018).
35 Li, L., Stoeckert, C. J., Jr. & Roos, D. S. OrthoMCL: identification of ortholog groups for eukaryotic genomes. *Genome Res* **13**, 2178-2189, doi:10.1101/gr.1224503 (2003).

36 Wallace, I. M., O'Sullivan, O., Higgins, D. G. & Notredame, C. M-Coffee: combining multiple sequence alignment methods with T-Coffee. *Nucleic Acids Res* **34**, 1692-1699, doi:10.1093/nar/gkl091 (2006).
37 Li, W. & Godzik, A. Cd-hit: a fast program for clustering and comparing large sets of protein or nucleotide sequences. *Bioinformatics* **22**, 1658-1659, doi:10.1093/bioinformatics/btl158 (2006).
38 Wang, Y. P. *et al.* MCScanX: a toolkit for detection and evolutionary analysis of gene synteny and collinearity. *Nucleic Acids Research* **40**, doi:10.1093/nar/gkr1293 (2012).
39 Slater, G. S. & Birney, E. Automated generation of heuristics for biological sequence comparison. *BMC Bioinformatics* **6**, 31, doi:10.1186/1471-2105-6-31 (2005).
40 Edgar, R. C. MUSCLE: multiple sequence alignment with high accuracy and high throughput. *Nucleic Acids Research* **32**, 1792-1797, doi:10.1093/nar/gkh340 (2004).
41 Krzywinski, M. *et al.* Circos: an information aesthetic for comparative genomics. *Genome Res* **19**, 1639-1645, doi:10.1101/gr.092759.109 (2009).
42 Langmead, B. & Salzberg, S. L. Fast gapped-read alignment with Bowtie 2. *Nat Methods* **9**, 357-359, doi:10.1038/nmeth.1923 (2012).
43 Kim, D., Langmead, B. & Salzberg, S. L. HISAT: a fast spliced aligner with low memory requirements. *Nat Methods* **12**, 357-360, doi:10.1038/nmeth.3317 (2015).
44 Pertea, M. *et al.* StringTie enables improved reconstruction of a transcriptome from RNA-seq reads. *Nat Biotechnol* **33**, 290-295, doi:10.1038/nbt.3122 (2015).
45 Yang, Z. *et al.* Comparative transcriptome analyses reveal core parasitism genes and suggest gene duplication and repurposing as sources of structural novelty. *Mol Biol Evol* **32**, 767-790, doi:10.1093/molbev/msu343 (2014).
46 Finn, R. D. *et al.* HMMER web server: 2015 update. *Nucleic Acids Res* **43**, W30-38, doi:10.1093/nar/gkv397 (2015).
47 Walker, J. C. Structure and function of the receptor-like protein kinases of higher plants. *Plant molecular biology* **26**, 1599-1609 (1994).
48 Torii, K. U. Receptor kinase activation and signal transduction in plants: an emerging picture. *Curr Opin Plant Biol* **3**, 361-367 (2000).
49 Shiu, S. H. & Bleecker, A. B. Expansion of the receptor-like kinase/Pelle gene family and receptor-like proteins in Arabidopsis. *Plant Physiol* **132**, 530-543, doi:10.1104/pp.103.021964 (2003).
50 Wu, Y. *et al.* Genome-Wide Expression Pattern Analyses of the Arabidopsis Leucine-Rich Repeat Receptor-Like Kinases. *Molecular plant* **9**, 289-300, doi:10.1016/j.molp.2015.12.011 (2016).
51 Shiu, S. H. *et al.* Comparative analysis of the receptor-like kinase family in Arabidopsis and rice. *The Plant cell* **16**, 1220-1234, doi:10.1105/tpc.020834 (2004).
52 Zou, C. & Bleecker, A. B. Receptor-like kinases from Arabidopsis form a monophyletic gene family related to animal receptor kinases. *Proceedings of the National Academy of Sciences of the United States of America* **98**, 10763-10768, doi:10.1073/pnas.181141598 (2001).

53 Gomez-Gomez, L. & Boller, T. FLS2: an LRR receptor-like kinase involved in the perception of the bacterial elicitor flagellin in Arabidopsis. *Molecular cell* **5**, 1003-1011 (2000).
54 Clark, S. E., Running, M. P. & Meyerowitz, E. M. CLAVATA1, a regulator of meristem and flower development in Arabidopsis. *Development* **119**, 397-418 (1993).
55 Pond, S. L., Frost, S. D. & Muse, S. V. HyPhy: hypothesis testing using phylogenies. *Bioinformatics* **21**, 676-679, doi:10.1093/bioinformatics/bti079 (2005).
56 Smith, M. D. *et al.* Less Is More: An Adaptive Branch-Site Random Effects Model for Efficient Detection of Episodic Diversifying Selection. *Molecular Biology and Evolution* **32**, 1342-1353, doi:10.1093/molbev/msv022 (2015).
57 Wertheim, J. O., Murrell, B., Smith, M. D., Kosakovsky Pond, S. L. & Scheffler, K. RELAX: detecting relaxed selection in a phylogenetic framework. *Mol Biol Evol* **32**, 820-832, doi:10.1093/molbev/msu400 (2015).
58 Thompson, J. D., Higgins, D. G. & Gibson, T. J. CLUSTAL W: improving the sensitivity of progressive multiple sequence alignment through sequence weighting, position-specific gap penalties and weight matrix choice. *Nucleic Acids Research* **22**, 4673-4680 (1994).
