# Supplementary material for: Large-scale gene losses underlie the genome evolution of parasitic plant Cuscuta australis
Source: Nat Commun. 2018 Jul 11;9:2683. doi: 10.1038/s41467-018-04721-8 (PMC6041341; doi:10.1038/s41467-018-04721-8)
Supplement: Supplementary file 3 — Description of Additional Supplementary Files [file 41467_2018_4721_MOESM3_ESM.pdf]

Description of Additional Supplementary Files

File Name: Supplementary Data 1
Description: Supplementary Data 1a. Gene family expansion and contraction; Supplementary Data 1b. Number of orthogroup genes in each species; Supplementary Data 1c. Gene loss identified using Arabidopsis genes as reference; Supplementary Data 1d. Tissue-specific expression levels of the orthogroup genes in *Solanum lycopersicum*, *Ipomoea nil*, *and Cuscuta pentagona*

File Name: Supplementary Data 2
Description: Supplementary Data 2a. GO enrichment of the orthogroups whose orthologous members are conserved in 7Ref-Species but lost in both *Cuscuta australis* and *Utricularia gibba*. Supplementary Data 2b. GO enrichment of the orthogroups whose orthologous members are conserved in 7Ref-Species and *Utricularia gibba*, but lost in *Cuscuta australis*. Supplementary Data 2c. GO enrichment of the orthogroups whose orthologous members are conserved in 7Ref-Species and in *Cuscuta australis*, but lost in *Utricularia gibba*. Supplementary Data 2d. GO enrichment of the principally expressed genes in *Cuscuta australis* haustoria. Supplementary Data 2e. GO enrichment of positively selected genes in *Cuscuta australis*. Supplementary Data 2f. GO enrichment of relaxed purifying selection genes in *Cuscuta australis*. Supplementary Data 2g. GO enrichment of genes in expanded gene families in *Cuscuta australis*.

File Name: Supplementary Data 3
Description: Supplementary Data 3a. Functional annotations of pseudogenes. Supplementary Data 3b. R genes in *Cuscuta australis* and 7Ref-Species. Supplementary Data 3c. TPS genes in *Cuscuta australis* and 7Ref-Species. Supplementary Data 3d. P450 genes in *Cuscuta australis* and 7Ref-Species. Supplementary Data 3e. RLK genes in *Cuscuta australis* and 7Ref-Species. Supplementary Data 3f. Functional annotations of principally expressed genes in *Cuscuta australis* haustoria.

File Name: Supplementary Data 4
Description: Presence/absence of plastid tRNA, rRNA, and protein-coding genes in Nicotiana tabacum, Ipomea nil, four Cuscuta spp., and two root parasitic plants Striga hermonthica and Orobanche cumana.

File Name: Supplementary Data 5
Description: Copy numbers of Copia and Gypsy families in *Cuscuta australis* and *Ipomoea nil*.
